# Peripheral Nerve Regeneration: A Current Perspective

**Published:** 2009-10-12

**Authors:** Christine Radtke, Peter M. Vogt

**Affiliations:** Department of Plastic, Hand and Reconstructive Surgery, Hannover Medical School, 30625 Hannover, Carl-Neuberg Strasse 1, Hannover, Germany

## Abstract

**Objective:** Nerve regenerative is a complex problem and cell therapy strategies are being developed to enhance axonal regeneration. One approach is to transplant peripheral myelin–forming cells (Schwann cells or olfactory ensheathing cells) that can secrete neurotrophic factors and participate in remyelination of regenerated axons. The objectives of this report are to first review the basic regeneration properties of myelinated axons. Next, to review studies that show functional improvement after transplantation of peripheral myelinating cells in the injured spinal cord. The final objective is to review recent studies using this approach as an adjunct cell therapy for microsurgical repair of peripheral nerve. **Methods:** Schwann cells and olfactory ensheathing cells were transplanted into injured spinal cord and peripheral nerve. In the microsurgical repair studies, rat sciatic nerves were repaired with epineural sutures (10.0). Olfactory ensheathing cells were transplanted in the experimental group at the time of repair. Histological and behavioral assessment was carried out at 5 weeks postsurgery. **Results:** Experimental transplantation of olfactory ensheathing cells at the time of microsurgical repair of peripheral nerve leads to increased axonal regeneration across the repair site and improved functional outcome. **Conclusions:** Olfactory ensheathing cells can integrate and participate in neural repair in both spinal cord and peripheral nerve. They promote axonal sprouting and contribute to remyelination associated with appropriate axon nodal sodium channel clustering necessary for proper impulse conduction. These experimental observations suggest that adjunct cell transplantation with microsurgical repair should be considered as a possible tool in peripheral nerve repair.

Damage to peripheral nerve fibers often results in axonal loss and demyelination followed by regeneration and remyelination under optimal conditions with the possibility of at least some functional recovery. In the central nervous system (CNS), unassisted axonal regeneration is at best rare. Much recent work now indicates that this difference between peripheral nervous system (PNS) and CNS axonal regeneration is the result of both permissive factors present in the PNS and active inhibitory factors present in the CNS. Indeed, knowledge of these differences has encouraged research to develop novel strategies to enhance axonal regeneration. After nerve repair, clinical results are often disappointing and experimental approaches to enhance functional recovery are on their way. These include focal application of neurotrophic factors, blockade of axonal regeneration inhibitory molecules, and cell transplantation. In this article, we review the basic organization of peripheral axons and discuss current experimental strategies using transplantation of peripheral myelin–forming cells to enhance axonal regeneration and remyelination both in the spinal cord and in microsurgical nerve repair.

## ANATOMICAL AND PHYSIOLOGICAL BASES

### Organization of myelinated peripheral nerve fibers

**Figure 1:**
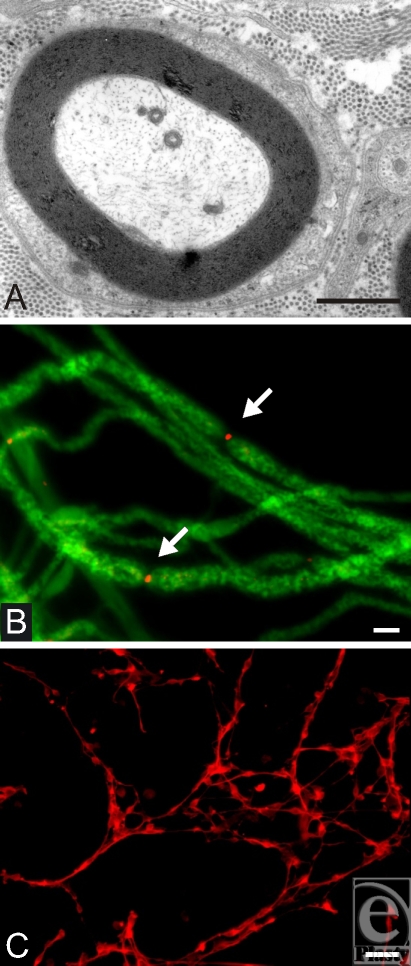
(A) Electron micrograph demonstrating typical peripheral myelinating axon in a cross section; note the densely packed myelin sheaths around the axon. In addition, characteristic extracellular collagen can be seen in the outer margins. Scale bar = 2 μm. (B) Dissociated single axons with neurofilament (green) and sodium channel staining for sodium channel subtype Nav 1.6 (red). Accumulation of Nav 1.6 is indicative for nodes of Ravier (arrows). Scale bar = 4 μm. (C) Subconfluent culture of olfactory ensheathing cells stained with p75^*NGFR*^ demonstrating characteristic morphology with bipolar shape. Scale bar = 40 μm.

Peripheral myelinated axons (Fig1A) are myelinated by Schwann cells, which form a single myelin segment or internode. This is in contrast to myelin in the CNS, which is formed by the oligodendrocyte and sends out several processes to form numerous myelin segments. A basal lamina with laminin on its internal surface surrounds each myelinated axon in the PNS. Slower conducting nonmyelinated axons in the peripheral nerve responsible for pain and temperature (C-fibers) are associated with Schwann cells, which surround groups of these fibers but do not form myelin. Thus, a continuous basal lamina tube surrounds individual myelinated fibers and groups of nonmyelinated fibers for the entire length of the nerve. Another unique feature of peripheral nerve as opposed to central white matter is the deposition of extracellular collagen in the endoneurial compartment.

The node of Ranvier is the short segment of axon between adjacent Schwann cells and is the site of action potential generation. The internode distance of a large myelinated axon can be as long as a millimeter, but the node of Ranvier is typically only several microns in length (Fig 1B). The action potential of myelinated axons is generated at the relatively narrow node of Ranvier and “skips” from node to node, providing for saltatory conduction.

Much work indicates that sodium channels, the molecular batteries generating the action potential, are present at the node in relatively high concentration. Experiments using radiolabeled saxitoxin have estimated the relative density of sodium channel at the mammalian node and internode.^1^ Data suggest that nodal sodium channel density is about 1000 to 2000 sodium channels/μm^2^ of the nodal membrane and the internodal membrane has a sodium channel density μof about 25/μm^2^.^2^ Shrager et al^3^ used a loose patch-clamp technique and estimated an internodal sodium channel density of only 20 to 25/μm^2^, which is insufficient to generate an action potential. There are numerous sodium channel subtypes with different electrical properties.^4^ It has been established that the sodium channel subtype Nav 1.6 is the tetrodotoxin-sensitive, kinetically fast channel present at the normal node of Ranvier of peripheral nerve fibers (Fig 1B, arrows).^5^ The very high density of voltage-gated sodium channels at the node is important because synchronous activation of these channels provides substantial current to ensure the efficacy of activation of the next set of nodes and high-fidelity saltatory conduction. Moreover, for fast conducting regenerated myelinated axons to be functional, they must recapitulate appropriate nodal and myelin architecture.

## Wallerian degeneration

When the axon is severed by nerve injury, the axon diebacks a millimeter or two from the injury site and the distal segment degenerates, a feature known as Wallerian degeneration.^6^ The myelin debris is phagocytized by macrophages. While the axon segment distal to the injury site degenerates, the Schwann cells proliferate typically within the basal lamina and form a column of Schwann cells or band of Büngner. This Schwann cell column is an indispensable pathway for directed axonal regeneration. Axons regenerate within these basal lamina tubes to reach motor or sensory targets.^7^ If regenerating axons do not grow within this environment, but through the endoneurial space within the connective tissue, they stop regenerating and do not reach target. Thus, the Schwann cell column provides an important permissive environment for axonal regeneration.

## REGULATORY MECHANISMS OF REPAIR: GROWTH FACTOR AND RECEPTOR EXPRESSION

### Upregulation of Schwann cell NGF and the p75 NGF receptor

Nerve growth factor (NGF) is produced by target tissues both of sympathetic neurons and of sensory nerves.^8^ mRNA expression of NGF in Schwann cells of normal peripheral nerve is very low, but after axotomy, NGF and the low-affinity p75 NGF receptor, p75^*NGFR*^, are considerably upregulated on Schwann cells.^9^–^11^ Interestingly, Schwann cells in the distal degenerating nerve segment downregulate NGF and p75^*NGFR*^ expression after contact with regenerating axons.^10^ It has been hypothesized that Schwann cell–derived NGF is secreted and links Schwann cell surface p75 receptors to NGF receptors on the regenerating axons. Moreover, it has been suggested that the p75 receptor is then internalized in the regenerating axon and retrogradely transported back to the nucleus where it can influence gene expression associated with axonal regeneration. In addition, other neurotrophins such as brain-derived neurotrophic factor,^12^ ciliary neurotrophic factor,^13^ and basic fibroblast growth factor^14^ are upregulated in Schwann cells in the degenerating axon segment. These various neurotrophic factors can influence different functional classes of axons such as sensory and motor fibers, and research to better understand the timing of expression and precise role of these factors in axonal regeneration will be important.

## ANATOMICAL MECHANISMS OF REPAIR

### Axonal sprouting and regeneration

After a delay of a day or two, the proximal stump of the cut nerve gives rise to axonal sprouts that will extend either on the surface of Schwann cells or to the inner laminin-rich surface of the basal lamina of the Schwann cells columns (bands of Büngner). If the proximal and distal segments of the nerve appose each other, regenerating axons may grow through these Schwann cell “scaffolds” and make contact with peripheral targets with the possibility of some functional recovery. Typically, several axonal sprouts issue from a single regenerating fiber and grow within a single Schwann cell column, but after target contact, all but one of the sprouts dieback leaving a single fiber making contact with a peripheral target. The regenerated fiber is then myelinated by Schwann cells within the tube and conduction velocity is increased to appropriate levels.^15^ In general, regenerated and remyelinated axons have shorter internodes, thinner myelin, and smaller axonal diameters, but they can achieve rapid conduction.^16^

If the regenerating axon sprouts do not reach and elongate through the trophic distal Schwann cell tube, they will grow in a more random manner and can form a neuroma.^7^ This is often the case after traumatic peripheral nerve injury, including limb amputation. The axonal sprouts within the bulbous neuroma show increased mechanosensitivity and chemosensitivity.^17^ These hyperexcitability changes are often associated with paresthesia and painful events including phantom limb in the case of limb amputation. Interestingly, sensory neurons show distinct changes in sodium^18^ and potassium^19^ channels on their cell bodies. These changes are associated with ectopic or spontaneous firing of action potentials from the sensory neuronal cell bodies.^20^ Chronic focal application of NGF to the cut nerve end where the neuroma forms reduces these changes.^18^,^19^ Thus, the excitability changes following nerve injury can originate not only from the cut end of the nerve or the neuroma head but also from changes in excitability in the sensory neuronal cell body in the dorsal root ganglia. Moreover, retrograde transport of NGF may play a role in stabilizing ion channel organization of the sensory axon and cell body. NGF is normally produced in β-keratinocytes in the skin and may provide an ambient level of NGF to stabilize excitability of primary afferent cutaneous fibers.

## CELLULAR AND SURGICAL INTERVENTIONS TO IMPROVE REPAIR

### Transplantation of peripheral myelinating cells to encourage axonal regeneration and remyelination in the spinal cord

Long-tract axons in the mammalian spinal cord do not normally regenerate for an appreciable distance within the denervated host tract after they are transected. This is likely because of a lack of the permissive environment that the Schwann cell provides in peripheral nerve^21^ and the presence of active inhibitory factors that elicit growth cone collapse such as the NOGO molecule present on CNS (oligodendroctye) myelin.^22^ Knowledge of the potential of peripheral nerve regeneration has lead to several experimental approaches that have been reported to improve elongative regeneration of axons in the transected mammalian spinal cord. These include blockade of inhibitory proteins on glial cells^22^,^23^ and introduction of neurotrophic factor–enhanced peripheral nerve bridges.^24^

Recent attention has focused on transplants of cultured olfactory ensheathing cells (OECs) into injured spinal cord^25^,^26^ and into nerve bridges in the spinal cord^27^ to enhance regeneration. OECs have several unique properties, which provide a rationale for their potential to enhance CNS axonal regeneration. They are specialized cells that support axons that leave the olfactory epithelium and project through the PNS into the olfactory bulb of the CNS; they are pluripotential cells that can show Schwann cell or astrocyte-like cell properties.^28^ Morphology of OECs (Fig 1C) is similar to myelinating Schwann cells, with characteristic spindle-shaped cell body and bipolar appearance of cell shape. The typical marker is p75^*NGFR*^. Interest has focused on these cells because olfactory epithelial neurons are continuously replaced and regenerate peripheral axons in the adult.^29^,^30^ It has been reasoned that the unique properties of OECs may allow them to guide and enhance regenerating CNS axons through a normally growth inhibitory environment.^25^,^31^

OEC transplantation can enhance regeneration of transected spinal cord axons and improve forepaw reaching behavior^25^ and remyelinate demyelinated axons in the spinal cord.^32^,^33^ Following transplantation of either OECs or Schwann cells into rat spinal cord following transection of the dorsal funiculus, the regenerating ascending sensory axons displayed stable conduction properties with regard to conduction velocity and frequency-response properties.^33^,^34^ Transplantation of Schwann cells into transected spinal cord white matter also leads to long-tract axonal regeneration.^33^,^34^ These results indicate that the regenerated spinal cord axons reconstitute electrophysiological function, an important requirement for an interventional therapy to enhance axonal regeneration after spinal cord injury. Therefore, although the number of regenerated axons induced by cell transplantation of OECs or Schwann cells is limited, a rapidly and securely conducting new information line is established that may contribute to the observed behavioral recovery of function. Thus, creation of a peripheral nerve–like environment in the spinal cord by peripheral myelin–forming cell transplantation may enhance functional recovery.

Although endogenous Schwann cells play an important role in the regeneration of peripheral nerve, transplantation of Schwann cells, or OECs, could in principle assist the regenerative process. For example, if severed nerve is surgically reapposed, it may take time for the endogenous Schwann cells to appropriately differentiate and organize to provide an optimal regenerative environment. Cultured Schwann cells transplanted to the apposition site could facilitate the regenerative process. Importantly, the newly formed nodes of Ranvier of the regenerated axons expressed sodium channel subtype Nav 1.6,^35^ the normal predominant nodal sodium channel. This indicates that engraftment of exogenous Schwann cells into injured nerve can reconstitute myelin and appropriate sodium channel organization necessary for proper impulse conduction.

## Transplantation of peripheral myelin–forming cells as an adjunct to microsurgical nerve repair

Although peripheral myelinating cells were first used to encourage axonal regeneration in the spinal cord, more recently, transplantation of OECs has been considered as an adjunct for peripheral nerve repair. The rationale is that they may provide a scaffold for axons to regenerate as well as trophic support and directional cues.^36^ Engraftment of OECs into axotomized facial nerve enhances axonal sprouting^37^,^38^ and, importantly, promotes recovery of vibrissae motor performance.^39^ In another study, the rate of eye closure was increased following OEC transplantation in a facial nerve lesion model.^40^ We transplanted Schwann cells^35^ and OECs^41^ into transected sciatic nerve and found that they integrate into the injury site and form peripheral myelin on the regenerated axons. Five weeks after transplantation, the nerves were studied histologically. GFP-expressing Schwann cells and OECs survived in the lesion and distributed longitudinally across the lesion zone. The internodal regions of individual teased living fibers were identified by GFP in the cytoplasmic and nuclear compartments of cells surrounding the axons. Immunostaining for sodium channel and Caspr (paranodal marker) revealed a high density of Nav 1.6 at the newly formed nodes of Ranvier, which were flanked by paranodal Caspr staining. These results indicated that the transplanted peripheral myelin–forming cells (Schwann cells and OECs) extensively integrate into transected peripheral nerve, form myelin on regenerated peripheral nerve fibers, and restore proper nodal structure in the injured PNS, indicating that they can contribute to local nerve repair.^41^

The next question was whether transplantation of peripheral myelinating cells at the time of microsurgical repair could improve functional outcome. In a recent article, we demonstrated that OECs transplanted at the time of microsurgical nerve repair enhanced nerve regeneration and functional outcome.^42^ Although axons normally dieback after injury for a short distance before they begin to regenerate, we found reduced dieback of the axons proximal to the transection site and an increased number of regenerated axons distal to the transection site. Interestingly, the microsuture-repaired nerves were thinner in diameter at the repair site than did the nerves that had the adjunct OEC transplants. Our current hypothesis is that the transplanted OECs are primed to produce neurotrophins and, therefore, can have an immediate effect on the injured axons. This may allow for less axonal dieback and earlier regeneration of the injured axons, thus allowing the regenerating axons to more effectively navigate across the nerve injury site before significant scar formation occurs.

## CONCLUSION

Experimental work indicates that peripheral nerve has a remarkable capacity to regenerate after injury. Reasons include the permissive environment produced by reactive Schwann cells in the distal nerve segment and the lack of axonal inhibitory proteins. Schwann cells not only provide a structural scaffold for regeneration by contributing to the bands of Büngner but also are a rich source of neurotrophic support, including NGF. When appropriate alignment of proximal and distal stumps of cut nerve occurs, the probability of correct nerve-target reestablishment is increased. When regenerating peripheral nerves are blocked from reacting target and neuroma formation occurs, the regenerating axonal sprouts become maladaptive and contribute to pain and paresthesias including phantom limb pain in the case of limb amputation. Several experimental strategies including focal neurotrophin treatment and cellular transplantation are being studied in the laboratory to optimize both surgical nerve repair and dysesthesias. The use of peripheral myelin–forming cells into areas of spinal cord injury results in improved functional outcome, and clinical studies are ongoing. More recent experimental work indicates that the transplantation of OECs into the site of microsurgical nerve repair leads to improved regeneration and functional outcome. An important challenge is to translate the recent advances in regeneration biology to novel surgical interventional approaches in the treatment of nerve injury.
